# The role of social value orientation in modulating vaccine uptake in the COVID-19 pandemic: A cross-sectional study

**DOI:** 10.1057/s41599-022-01487-9

**Published:** 2022-12-27

**Authors:** Sheena Moosa, Raheema Abdul Raheem, Aminath Riyaz, Hawwa Shiuna Musthafa, Aishath Zeen Naeem

**Affiliations:** The Maldives National University, Male’, Maldives

**Keywords:** Health humanities, Science, technology and society

## Abstract

COVID-19 vaccination is the only pharmaceutical measure available to control the pandemic and move past the current crisis. As such, the Maldives, a small island country, invested heavily on securing and vaccinating the eligible population through an intensive risk communication campaign to create awareness on vaccination benefits. This paper reports on the vaccine coverage after a year of COVID-19 vaccine being introduced into the country, based on data obtained from the Values in Crisis Survey – Wave Two among Maldivian adults (*n* = 497). The findings show a vaccine coverage of 94%, with only 2.2% of the respondents indicating they will not get vaccinated. No significant differences were observed by age, gender, income earning, educational status or residential area. No significant relationship was observed in vaccine behaviour and confidence in government, health sector and experts. Social value orientations, particularly conservation and self-transcendence value orientations determined positive vaccine behaviour (*r*_*s*_ = 0.180, *p* < 0.01 and 0.136 *p* < 0.01 respectively), yet conservation was the only predictor that contributed significantly to the regression model (*B* = 0.158, *p* < 0.01). The findings indicate that, despite the uncertainties around COVID-19 vaccinations, the prosocial value orientations were instrumental in achieving a high COVID-19 vaccine coverage. Further theoretical and conceptual exploration of vaccine behaviour in crisis situations is needed to inform future pandemic situations. The vaccination rollout and behaviour change strategies also need an examination of social value orientations in order to achieve a high coverage and sustain pro-vaccine behaviour post-pandemic.

## Introduction

Controlling a pandemic requires using all the tools available, and vaccines provide the most hopeful solution. Currently, ten COVID-19 vaccine products have been listed in the Emergency Use Listing (World Health Organization, 2021), while different jurisdictions have their local medicines and regulatory bodies approving the use of some of the vaccine products. The availability of vaccine products and immunization with COVID-19 vaccines became the priority strategy to mitigate the effects of the pandemic. Vaccination rollout, however, has been erratic across many countries due to limited production capacity, which has resulted in vaccine equity issues at a global level (Asundi et al., 2021).

Evidence suggests that availability and knowledge of a vaccine product do not automatically translate into vaccine uptake (Horne et al., 2015; Danchin et al., 2020). The prominent conceptualisations of behaviour such as the health belief model, information-motivation-behavioural skills model, theories of reasoned action and planned behaviour, and social cognitive theory draw on social and psychological theories (Armitage and Conner 2000; Reid and Aiken 2011). Furthermore, behaviour is often conceptualised as a point on a continuum of adopting a certain practice – such adoption is then influenced by internal individual factors as well as external environmental factors including media, social networks and social norms (Sutton 2005; Morris et al. 2012; Brewer et al. 2017; Viswanath et al. 2021). As such, a combination of thoughts, feelings, moral values, beliefs and worldviews may influence personal conclusions on vaccination (Yale Institute of Global Health 2020; Wiley et al. 2021).

Social value orientations act as the guiding moral principle for individual behaviour, and have been demonstrated to influence personal behaviour particularly at times of crisis (Moosa et al. 2021; Wolf et al. 2020). Schwartz (1992) posits that value orientations operate in a motivational continuum from the social focus to personal. He placed ten personal values into two higher order bipolar dimensions: self-transcendence versus self-enhancement and openness-to-change versus conservation. Self-transcendence and conservation value orientations are concerned with social outcomes by preserving cooperation. Particularly, it is observed that compliance to disease prevention and public health measures are higher in societies that have prosocial value orientations (Lake et al. 2021).

In addition to social values, trust in institutions is another social predictor of behaviour in a pandemic situation, and is an important determinant of public behaviour in times of crisis (Wolf et al. 2020). Mitra and colleagues (2016) note that individuals who have deep-rooted mistrust in the government are more hesitant to adopt government-recommended measures, including vaccination. Individuals whose economic status is negatively affected by the pandemic are also less likely to get vaccinated because of mistrust towards government policies (Piltch-Loeb et al. 2022). Trust in the vaccine, in manufacturers, and in health experts become important drivers in cases of a new vaccine such as COVID-19 vaccines that have been created and introduced in record time (Gesser-Edelsburg et al. 2018; Mesch and Schwirian 2015). As such, medical experts play an important role in advocacy for vaccine uptake (Lewandowsky et al. 2012). On the whole, successful coverage of the population with COVID-19 vaccines requires implementation of interventions and communications to build trust not only in vaccine products, but also trust in experts and the government.

Amidst the pandemic, in an attempt to achieve further control, the Maldives initiated vaccination campaigns, free for all residents (meeting specified technical criteria for safe inoculation), with vaccines approved for emergency use on 7 February 2021. Vaccine donations from other countries and the international COVAX facility supplemented government procurement (Ministry of Health 2021). The first rollout was using the vaccine ChAdOx1-S [recombinant] – Covishield and AstraZeneca COVID-19 AZD1222, followed by the now inactivated COVID-19 vaccine BIBP Sinopharm. In addition, mRNA vaccine, BNT162b2, Pfizer–BioNTech was supplied by the COVAX facility. By 9 January 2022, the Maldives reported a vaccine coverage of 67% (368,241 out of 546,399 resident population) with two doses (World Health Organization 2022). This coverage is somewhat lower than expected, based on the findings of Amir et al. (2021) that showed a high (86%) receptiveness towards COVID-19 vaccination in the Maldives.

While this is so, there is a limited understanding of the social drivers behind vaccine uptake, particularly when previous studies during the pandemic had shown only a moderate level of trust in government institutions and compliance to public health measures in the Maldives (Moosa et al. 2021). Models of behaviour change identify that vaccination behaviour is influenced by deep-rooted factors such as moral values, ideology and identity (Yale Institute of Global Health 2020). These, in turn, influence trust in institutions and experts that motivate positive attitudes and behaviour. Consistent with this proposition, during the pandemic it has been observed that societies with predominant prosocial value orientations are more compliant to public health measures (Wolf et al. 2020). The influence of values and norms during the COVID-19 pandemic have been studied using different conceptualisations. Agranov (2021) approaches this question through experimenting social norms and herding on vaccine uptake. Others have explored political ideologies and how trust in government impacts vaccination behaviour (Brewer et al. 2017; de Figueiredo et al. 2020; Kempthorne and Terrizzi 2021; Viswanath et al. 2021). Lake and colleagues (2021) studied the persuasiveness of public health messaging and social value orientations on vaccination behaviour. These studies indicate that social value orientations play an important role in people’s behaviour in times of crisis such as the COVID-19 pandemic. However, the relationship between social norms with behaviour is studied often with a focus on a political ideology rather than social value orientations (Kempthorne and Terrizzi 2021). While the work of Lake and colleagues (2021) examined the association between social value orientation and vaccine uptake along with social distancing behaviour, the focus was on value-expressive messaging.

**Fig. 1:**
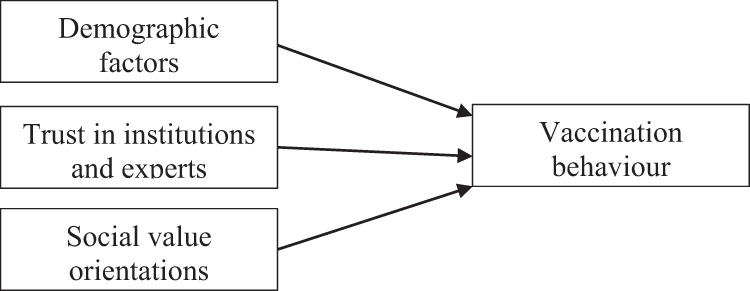
Conceptual framework on determinants of vaccine behavior. It shows the relationship between theindependent and dependent variables studied.

Drawing from the existing work, this study aims to contribute to the emerging evidence on the role of social value orientations on vaccine behaviour in pandemic crisis situations. This study conceptualises that, during a pandemic, social value orientations are one of the independent determinants of vaccination behaviour in addition to levels of trust and other demographic identity factors (see Fig. 1).

## Methods

This paper draws on the data from the Values in Crisis Survey – Wave Two using a cross-sectional survey design. Data was collected during October to December 2021 in the Maldives from the cohort of randomly selected sample that completed the Values in Crisis Survey – Wave One in May 2020 (Riyaz et al. 2020).

The sampling strategy adopted for the Values in Crisis Survey is a multi-cluster population proportionate random sample from urban and rural communities of the Maldives, stratified by gender and age groups. After data cleaning, the final sample for this study included 497 (*n* = 185 urban, *n* = 312 rural) respondents with 46% males and 54% females, and 45.7% in the age group 18–34 years, 50.7% in 35–64 years and 3.6% in 65+years, representative of the local population of the Maldives.

### Measures of social value orientations

The analysis draws on the personal values questionnaire (PVQ-21) items included in the survey instrument for psychological measurement of values in the Schwartz values framework (Schwartz 2005, 2003). The respondents rated their response using a six-point scale ranging from 1 (very much like me) to 6 (not like me at all) for each item. For the analysis, all items were reverse scored: higher numbers would indicate greater agreement with the item. The ten personal values that are identified in this framework are placed into two higher order bipolar dimensions: openness-to-change versus conservation, and self-transcendence versus self-enhancement (for an overview see Schwartz 2012). In the analysis, these higher order bipolar dimensions are used as the social value orientations. The higher order values were computed by averaging participants’ responses to the items from the values that constitute it.

### Data cleaning

No missing data was identified for the 21 items on the PVQ-21 scale. Pattern responding on the PVQ-21 scale was flagged by identifying the participants who responded with the same response for more than 16 items. One-hundred and nineteen participants (19.3% of the total sample) were found to have pattern-responded and they were removed from the analysis as recommended in Schwartz’s instructions (Schwartz 2005). Hence, the final data set consists of 497 mnparticipants (*n* = 185 urban, *n* = 312 rural; 227 males and 270 females).

### Internal reliabilities

The reliability for this data set for all higher-order values was acceptable (α > 0.6). However, the reliability for the basic values was found to be poor, and at an unacceptable level for two values (self-direction and power). The low internal reliability can be expected as few items are used to measure each of the ten values, and items for each are chosen that encapsulate the different conceptual components in order to increase the breadth of the meaning of values, rather than choosing items that have similar meanings (Schwartz 2003).

### Computing value scores

Schwartz’s instructions for the PVQ-21 were used to compute the value scores (Schwartz 2003, 2005). Participants’ responses to the items that constitute the values were averaged to compute a score for each of the ten basic and higher order values. Corrected centred values were generated by centring each individual’s responses to their mean response for all 21 items (Schwartz 2005). This method of centring of value indices at the ‘within-individual level’ diminishes the effects of scale use response (respondent’s tendency to locate their responses on specific parts of the scale) and allows the generation of scores that measure the relative (instead of absolute) importance of values to the person (Schwartz 1996). Higher order values scores were generated by averaging participants’ response to the items that constitute the values:

- Openness to change: items 1, 10, 11, 6, 15, 21;
- Conservation: items 5, 9, 14, 7, 16, 20;
- Self-enhancement: items 2, 4, 17, 13; and
- Self-transcendence: items 3, 8, 12, 18, 19 (see Schwartz (2005, 1996) for detailed description).

### Other measures

The analysis also uses data from the items on the public confidence in the government, health sector, scientific experts and public broadcasters using single item questions to rate the respondent’s level of trust on each of these variables measured on a Likert scale 1 to 4 (1 = A great deal, 2 = Quite a lot, 3 = Not very much, 4 = None at all). All items were also reverse-scored.

The item on attitude towards vaccine uptake is used with responses numerically coded for the (1 = I will definitely not get vaccinated, 2 = I will probably not get vaccinated, 3 = I will probably get vaccinated, 4 = I will definitely get vaccinated, 5 = I already got vaccinated or have an appointment, 6 = I want to get vaccinated, but I cannot be due to health issues). For the analysis, response 6 was recoded into 2, to indicate a higher motivation to uptake of vaccination.

### Quantitative analysis

Percentage distribution of vaccine uptake, mean public confidence ratings, and social value orientations are presented by gender, age group, education status, economic status and urban rural geographic categories of the sample. To explore the relationships between vaccine uptake and social value orientations as well as perceptions of public confidence, correlation tests were done after converting the categorical responses to ordinal data on vaccine uptake and public confidence response. Further, a multivariate linear regression was performed to explore whether these variables determine the vaccine uptake.

## Results

The results indicated a high vaccine coverage in this sample with 97.8% having already received the vaccine or indicating they will get vaccinated, and only 2.2% indicating that they will not get vaccinated. No demographic factors were found to be significantly associated with vaccine uptake (see Table 1).

**Table 1:** Vaccine behaviour by age, gender, residential area, income earning and education status.

	Vaccine behaviour											
	I will not get vaccinated		I will probably not get vaccinated		I will probably get vaccinated		I will get vaccinated		I already got vaccinated or have an appointment		Total	Pearson Chi-square (Sig 2-tailed)
Factors	*N*	%	*N*	%	*N*	%	*N*	%	*N*	%	*N*	*χ*² (*p*)
Sex												7.52 (0.111)
Female	0	0	4	1.5	1	0.4	4	1.5	261	96.7	270	
Male	4	1.8	3	1.3	3	1.3	1	0.4	216	95.2	227	
Age group												5.99 (0.648)
18–34 years	3	1.3	4	1.8	3	1.3	4	1.8	213	93.7	227	
35–64 years	1	0.4	3	1.2	1	0.4	1	0.4	246	97.6	252	
65+ years	0	0	0	0	0	0	0	0	18	100	18	
Residential area												5.67 (0.225)
Urban	3	1.6	3	1.6	3	1.6	1	0.5	175	94.6	185	
Rural	1	0.3	4	1.3	1	0.3	4	1.3	302	96.8	302	
Income												2.78 (0.595)
No income	1	0.7	3	2	1	0.7	3	2	142	94.7	150	
Earns income	3	0.9	4	1.2	3	0.9	2	0.6	335	96.5	347	
Education												8.01 (0.425)
Primary and lower	1	0.9	3	2.8	0	0	2	1.9	100	94.3	106	
Secondary or higher	1	0.9	0	0	2	1.8	2	1.8	107	95.5	112	
Graduate	2	0.7	4	1.4	2	0.7	1	0.4	270	96.8	279	

Public confidence in institutions was moderate to low. Over half of the respondents answered ‘none at all’ or ‘not very much’ to the questions on confidence in the government (57.9%), country’s health sector (55.1%), and public service broadcasters (61.6%). However, the majority of the respondents (65%) indicated having ‘quite a lot or great deal’ of confidence in the country’s health experts. Regarding social value orientations, participants had higher mean scores for the higher order value of conservation (*M* = 5.16, *SD* = 0.694) over openness to change (*M* = 4.48, *SD* = 0.802). They also had higher mean scores for self-transcendence (*M* = 5.25, *SD* = 0.679) compared to self-enhancement (*M* = 3.96, *SD* = 0.922).

**Fig. 2:**
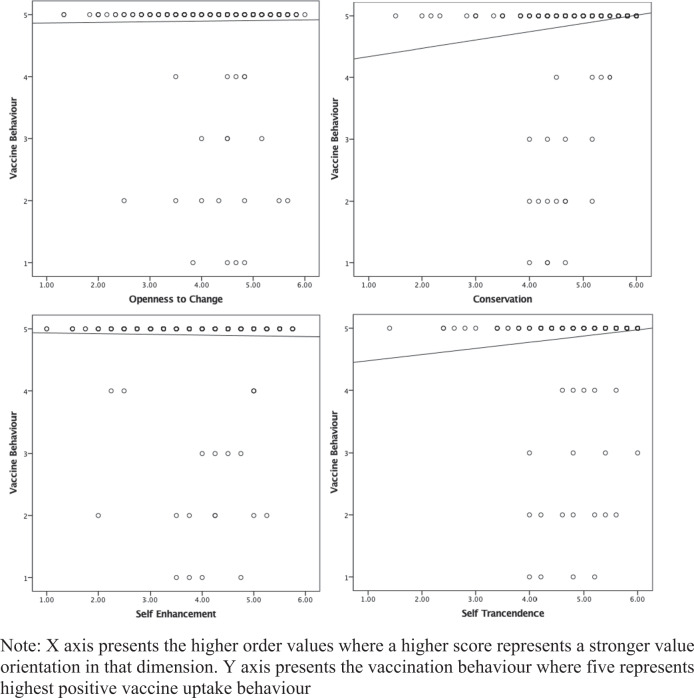
Relationship between vaccine behaviour scores and higher order values scores. They show the relationshipbetween these four higher-order values and vaccine behaviour.

The association of vaccine uptake with public confidence in institutions and social value orientations were analysed using Spearman’s Correlation (see Table 2). None of the factors of public confidence were found to be significantly associated with vaccine uptake. Significant positive associations were found between vaccine uptake and the basic values: security, conformity and benevolence; and a negative association was observed between power and vaccine uptake (see Table 2). Significant positive associations were also found between vaccine uptake and higher order values, namely conservation and self-transcendence (*r*_*s*_ = 0.180, *p* < 0.01 and 0.136 *p* < 0.01 respectively). Figure 2 presents the scatterplot of the association between the higher-order values and vaccination behaviour.

**Table 2:** Relationship between social value orientation and vaccine behavior.

	Spearman’s *(r*_*s)*_	*p*
Public confidence in the		
Government	−0.01	0.828
Health sector	−0.021	0.644
Public service broadcasters	0.029	0.515
Scientific experts	0.047	0.298
Basic Values		
Achievement	−0.043	0.335
Benevolence	0.095^a^	0.035
Conformity	0.117^b^	0.009
Hedonism	0.035	0.442
Power	−0.097^a^	0.031
Security	0.118^b^	0.009
Self-direction	−0.064	0.155
Stimulation	−0.046	0.306
Tradition	0.069	0.126
Universalism	0.068	0.131
Higher Order Values		
Conversation	0.180^b^	0
Openness to change	0.024	0.587
Self-enhancement	−0.033	0.463
Self-transcendence	0.136^b^	0.002

^a^Correlation is significant at the 0.05 level (2-tailed).

^b^Correlation is significant at the 0.01 level (2-tailed).

A multivariate linear regression was carried out to investigate the higher order values that determined participants’ vaccine uptake, whilst controlling for demographic factors. The model was a significant predictor of vaccine uptake (*F* = (9487) 3.184, *p* = 0.001, *R* = 0.236, *R*^*2*^ = 0.056), but only conservation was found to be the only predictor that contributed significantly to the model (*B* = 0.158, *p* = 0.004, 95% CI 0.050, 0.267).

## Discussion

The vaccine uptake during the pandemic of COVID-19 in the study context show some divergence to the common recognised drivers. Bish and Michie (2010) reported that demographic variables such as age, gender and education status affected vaccine uptake behaviour. However, the current study did not find any significant association by demographic factors. This observation may be attributable to the policy on free vaccination for all residents irrespective of their income, education or social status (Ministry of Health 2021). While, urban areas often have improved access to education and work, the lower vaccine uptake observed in the urban areas compared to rural areas suggest other social drivers, such as confidence in the institutions determining this difference. There is evidence that in the pandemic context in which COVID-19 vaccination is implemented, other drivers such as confidence in the institutions becomes the important driver of compliance with public health measures (Gesser-Edelsburg et al. 2018; Wolf et al. 2020; Larson et al. 2015).

Confidence in public institutions, health sector and health experts are proposed as important drivers of adherence to public health measures in the pandemic, including recommendations of vaccinations (de Figueiredo et al. 2020). However, the current study does not support this proposition. The findings did not show any significant association between vaccine uptake and confidence in government, health sector or experts. This is perhaps a reflection of the moderate level of confidence in public institutions that was also observed early in the pandemic (Moosa et al. 2021). However, this aspect needs further exploration in future studies. The high level of vaccine coverage observed in this study suggests the existence of other drivers mediating vaccine uptake in the pandemic context in the Maldives. It also suggests other social drivers, such as social norms and values, perhaps play a more significant role in determining vaccine uptake in the pandemic context (Abdallah and Lee 2021; Agranov et al. 2021).

There is ample evidence that social value orientations determine public behaviour, particularly in the pandemic situation (Ibanez and Sisodia 2020; Leder et al. 2020; Wolf et al. 2020). Findings from the current research context in the early stages of the pandemic showed that the social value orientations of the society were prosocial, and though not particularly strong, leaned towards conservation and self-transcendence values such as benevolence, security, conformity, tradition and universalism (Moosa et al. 2021). It is proposed that when a society’s social value orientations conform to conservation and self-transcendence values, social responsibility and compromise is reflected in public behaviour (Ibanez and Sisodia 2020; Lake et al. 2021). Hence, a society that is prosocial is likely to have a higher uptake of COVID-19 vaccination, as confirmed by the findings of this study.

Previous studies have observed that, in the higher order values dimension of conservation versus openness to change, the Maldivian society tilts towards conservation (Moosa et al. 2021). This indicates stronger attachment to tradition, conformity to authority and security. In the COVID-19 situation, the conservation value orientation provides the environment for the government to implement orders that foster security successfully. This is confirmed by the finding that 96.7% of [x] had already had their COVID-19 vaccination or had made appointments at the time of the study. The predictive model of this study further demonstrates that conservation value orientation is the driver of vaccine uptake in the Maldivian society. However, this observation may be specific to the crisis situation and thus temporary. Theories of social value orientations suggest that, in crisis situations, people temporarily adjust their value orientations to the opportunities available in their environment as well as to life-changing events (Bardi et al. 2009). Accordingly, the view that the association between social value orientation and the vaccine behaviour is transitional needs further exploration as the pandemic evolves.

Additionally, Agranov and colleagues (2021) argue that herding effects also influences behaviour towards vaccine uptake – that is, the social norms become more important when expectations of others getting vaccinated is also high, as is expected in a prosocial society. However, some researchers argue that their findings do not evidence that communication of common behaviour in the society determine behaviour toward vaccine uptake among young adults (Sinclair and Agerstrom 2021). Other researchers have explored the dynamics of network structures of cyber communities and beliefs among these network communities that affect behaviour towards health interventions (Li et al. 2020, 2022). The findings suggest that the vaccine uptake in the pandemic situation is more complex, and the observations may be confounded by other situational factors relevant to the pandemic situation. This is particularly so with the protraction of the pandemic, which brings the risk that motivation for social outcomes wanes over time, resulting in lower uptake of vaccines (Lake et al. 2021). In this situation, it is likely that other individual level drivers such as perceptions of one’s susceptibility to and severity of the disease affect behaviour towards vaccination as proposed in the health belief model (Armitage and Conner 2000; Bish and Michie 2010; Reid and Aiken 2011). However, the current study is limited in its exploration of the effect of other factors such as policies of differential access to services for vaccinated and non-vaccinated persons on vaccine uptake. Furthermore, the study participants were delimited to only locals, and hence did not capture behaviour towards vaccination among foreign migrant residents in the country, limiting generalisation of the findings to the population.

While the findings provide strong evidence for social value orientations as drivers of vaccine uptake in the COVID-19 pandemic situation, they are limited to establishing the relationship rather than any causal associations. The contribution from this study needs further work and testing, particularly as the discourse on social value orientation in crisis situations suggests that the findings may be limited to the specific crisis of the pandemic and may change if tested in the peacetime. Another future area of research is expanding the definition of social value orientation to capture the social values of cyber communities and relevance of current theories and measurements to these community networks, which is particularly important in this era of infodemics and disinformation. A number of methodological innovations in data science such as the Markov clustering algorithm and fusion engines are emerging and could assist in carrying out such further study. (Li et al. 2022).

Furthermore, the effect of policies on movement and travel restrictions for unvaccinated people that introduces inequity are likely to have influenced vaccine uptake, irrespective of the value orientation and trust in institutions (Tanner and Flood 2021). There is growing concern that vaccine mandates may not only affect health equity but carries the risk of widening socioeconomic disparities among populations (Gellert and Gellert 2021). Hence, further research is needed post-pandemic to study the impact of the vaccine mandates on the attitudes and behaviour towards future vaccination and social equity among the population.

## Conclusion

The findings indicated an association of social value orientation as a contributor to vaccine uptake in the pandemic situation and needs further exploration in non-crisis situations for validation as a predictor of vaccine uptake. Studies of behaviour towards vaccination need further refinement in theoretical construction and conceptualisation of vaccine behaviour in post-pandemic situations because of the continued evolution of the vaccination regime for COVID-19 with the protraction of the pandemic, and the vaccine disinformation that is continuing to emerge. It is recommended that such exploration extend the scope to cyber communities and technological evolutions and methodological innovations. The vaccination rollout and strategies also need further examination to learn lessons of achieving a high coverage and sustaining pro-vaccine behaviour in the future.

## Data Availability

All data generated or analysed during this study are included in this published article and its supplementary information files. The dataset from the survey questionnaire is deposited to the Values in Crisis data repository and available, but access restrictions apply. The dataset are also available from the corresponding author on reasonable request.
